# Mft1, identified from a genome-wide screen of the yeast haploid mutants, mediates cell cycle arrest to counteract quinoxaline-induced toxicity

**DOI:** 10.3389/fgene.2023.1296383

**Published:** 2024-01-12

**Authors:** Abdallah Alhaj Sulaiman, Dana E. Al-Ansari, Reem Ali, Mustapha Aouida, Dindial Ramotar

**Affiliations:** ^1^ Qatar Foundation, Division of Biological and Biomedical Sciences, College of Health and Life Sciences, Hamad Bin Khalifa University, Doha, Qatar; ^2^ National Heart and Lung Institute, Imperial College London, London, United Kingdom

**Keywords:** *Saccharomyces cerevisiae*, quinoxaline sensitive mutants, cell cycle arrest, genome-wide screening, drug resistance, antibacterial, antifungal, and antitumor activities

## Abstract

Quinoxaline is a heterocyclic compound with a two-membered ring structure that undergoes redox cycling to produce toxic free radicals. It has antiviral, antibacterial, antifungal, and antitumor activities. However, the biological functions that are involved in mounting a response against the toxic effects of quinoxaline have not been investigated. Herein, we performed a genome-wide screen using the yeast haploid mutant collection and reported the identification of 12 mutants that displayed varying sensitivity towards quinoxaline. No mutant was recovered that showed resistance to quinoxaline. The quinoxaline-sensitive mutants were deleted for genes that encode cell cycle function, as well as genes that belong to other physiological pathways such as the vacuolar detoxification process. Three of the highly sensitive gene-deletion mutants lack the *DDC1*, *DUN1*, and *MFT1* genes. While Ddc1 and Dun1 are known to perform roles in the cell cycle arrest pathway, the role of Mft1 remains unclear. We show that the *mft1Δ* mutant is as sensitive to quinoxaline as the *ddc1Δ* mutant. However, the double mutant *ddc1Δ mft1Δ* lacking the *DDC1* and *MFT1* genes, is extremely sensitive to quinoxaline, as compared to the *ddc1Δ* and *mft1Δ* single mutants. We further show that the *mft1Δ* mutant is unable to arrest in the G2/M phase in response to the drug. We conclude that Mft1 performs a unique function independent of Ddc1 in the cell cycle arrest pathway in response to quinoxaline exposure. This is the first demonstration that quinoxaline exerts its toxic effect likely by inducing oxidative DNA damage causing cell cycle arrest. We suggest that clinical applications of quinoxaline and its derivatives should entail targeting cancer cells with defective cell cycle arrest.

## Introduction

Quinoxaline (QXN) is an organic heterocyclic compound from the class diazines, with the chemical formula (C_8_H_6_N_2_) (see structure Supplementary Figure S1A). QXN is prepared via a condensation reaction of 1,2-diaminobenzene with a 1,2-dicarbonyl compound (Pereira et al., 2015; Chauhan et al., 2020). The derivatives of QXN are produced through different reactions that include oxidation, reduction, and nitration, as well as catalytic and non-catalytic reactions (Sato and Katritzky, 2008; Buravchenko and Shchekotikhin, 2023). QXN and its derivatives have various biological activities such as anti-fungal, anti-viral and anti-bacterial (Chandrasekhar et al., 2010; Patel et al., 2016; Montana et al., 2020). These molecules also exhibit pharmacological applications in neurological disorders, cardiovascular diseases, diabetes and cancer (Heravi et al., 2009; El Newahie et al., 2019). QXN also exhibits anti-inflammatory characteristics as its derivatives can inhibit the production of pro-inflammatory cytokines and other molecules involved in the inflammatory response, making the QXN family of drugs potential candidates for the treatment of inflammatory diseases (Tariq et al., 2018). Despite the pharmacological potential and its wide applications for more than half a century, the exact underlying molecular mechanism of QXN biological activity is still unknown. Nonetheless, a very early study hinted that QXN derivatives can damage DNA (Suter et al., 1978).

It was several decades later that Cheng G. et al. (2015) used a derivative of QXN, quinoxaline 1, 4-di-*N*-oxides, to understand how this family of drugs induced its antibacterial effect on *Escherichia coli* (Cheng et al., 2015). The study revealed that the drug primarily induces the bacterial SOS response, that is, the induction of at least 20 genes encoding proteins involved in DNA damage and repair such as recA, uvrA, B, and umuD, C, as well as attenuating the cell division and cause oxidative stress (Cheng et al., 2015). The evidence revealed that upon entry the drug is redox activated and then diminished the viability of *E. coli* by causing oxidative DNA damage *via* the elevation of reactive oxygen species (Cheng et al., 2015). Thus, enhancing the effectiveness of these quinoxaline derivatives would imply targeting the DNA damage response systems such as preventing cell cycle arrest so that the cells are unable to effectively commit to repairing damaged DNA and hence accumulate unrepaired DNA lesions triggering cell death. Olaquindox, another derivative of QXN that is used as a livestock feed additive, has also been shown to induce the production of reactive oxygen species in human HepG2 cells causing oxidative DNA damage and arresting the cells in the S-phase (Li et al., 2017). Other QXN derivatives such as triazole-linked quinoxalines can recognize and bind to telomeric G-quadruplexes leading to the inhibition of breast cancer cell growth by arresting the cells at the G0/G1 phase (Hu and Lin, 2023).

One approach to understanding the mechanism of action of QXN is to take advantage of the available tools in the budding yeast *Saccharomyces cerevisiae* (Sulaiman et al., 2022; Mohanty et al., 2023; Sulaiman et al., 2023). These include a collection of ∼4,800 haploid mutants, each deleted for a single non-essential gene. The haploid mutant collection can be used for different applications such as understanding biological processes, response to environmental stresses, as well as understanding molecular mechanisms of drug actions (Aouida et al., 2004). In this study, we used the haploid mutant collection to identify gene-deletion mutants that would cause either resistance or sensitivity to QXN. We first used the parent strain of the mutant collection to establish the concentration of QXN that would kill 50% of the cells (IC_50_) and used a range of QXN concentrations to identify mutants from the collection that are either resistant or sensitive to the drug. Herein, we reported the isolation of 12 mutants from the entire collection that exhibited varying sensitivities to QXN, and none displaying resistance to the drug. Two of the QXN-hypersensitive mutants, deleted for the *DUN1* and the *DDC1* gene, were defective in the DNA damage response and the cell cycle arrest pathways, respectively, suggesting that QXN acts by damaging the DNA and leading to cell cycle arrest. Another QXN-hypersensitive mutant is defective for the *MTF1* gene. *MTF1* encodes a protein that belongs to the THO complex involved in RNA transaction, but has not been previously shown to play a role in the cell cycle arrest pathway. We showed that the *mft1Δ* mutants are unable to properly arrest cells in the S phase and lack the G2/M arrest in response to QXN. Moreover, when deleted in the *ddc1Δ* caused the resulting double mutant, *ddc1Δ* *mft1Δ* to be sensitized to the drug. The data imply that Mft1 has a function that is distinct from the Ddc1 protein in the cell cycle arrest pathway in response to QXN exposure. This is the first report demonstrating that the toxic effects induced by QXN trigger cell cycle arrest. Thus, cancer cells with defective cell cycle arrest are expected to be hypersensitive to QXN.

## Materials and methods

### Yeast strains and media

BY4741 wild-type strain was the background of the gene deletion library, which was obtained from EUROSCARF (Frankfurt, Germany) (Giaever et al., 2002). Regular YPD (Yeast extract-peptone-dextrose) media from FORMEDIUM CCM0105 or SD minimal media were used to grow the strains. Quinoxaline 99% was purchased from Sigma-Aldrich with a catalogue number of Q1603, dissolved in sterile water, and kept as 1 M stock solution at–20°C. The 1 M stock solution of QXN can be stored for up to 1 year without losing efficacy. Hydroxyurea was purchased from Sigma-Aldrich, dissolved in sterile distilled water, filter sterilized and stored at–20°C as a 2 M stock.

### Spot test analysis to establish the sensitivity of strain BY4741 to QXN

The parent strain BY4741 was grown overnight in YPD broth at 30°C, and then the OD_600_ was adjusted to 1.0. The stock was then serially diluted to 1:10, 1:20, 1:50, and 1:100 and then spotted onto YPD plates without and with different QXN concentrations of 9.5, 11.5, 13.5, and 15.5 mM. The plates were incubated at 30°C for 48 h, and the Image Lab Touch Software (BioRad) was used to document the results.

### Survival assay

To assess the survival of *S. cerevisiae* in response to QXN and determine the lethal concentration at 50% (IC_50_). The parent strain BY4741 was grown overnight, and then the OD_600_ was adjusted to 0.6. Yeast cells were treated with increasing concentrations of QXN for a period of 4 h, then spotted onto YPD plate and incubated for 48 h at 30°C. The colonies were counted using ImageJ software, and the survival plot was prepared using GraphPad Prism.

### High-throughput QXN screen

The yeast haploid mutant collection strains were arrayed as previously described (Aouida et al., 2004), except for using a 96-floating pin replicator operated by ROTOR HDA (Singer, United Kingdom). A total of 96 colonies were arrayed per solid YPD plate without or with the indicated concentration of QXN. The ROTOR HDA was set to pin each mutant once from the master plate onto the drug plate and ensure that the mutant was deposited onto the plates by using five pinnings on the same spot. The pin was changed between each drug concentration to prevent the drug from contaminating the master plate. The plates were incubated for 48 h at 30°C and photographed with a digital camera (Bio-Rad Chemi-Doc MP Imaging system 2000) to visually compare the growth of every mutant in the presence or absence of QXN (see illustration in Supplementary Figure S3).

### Spot test analysis to verify the QXN hypersensitive strains

The QXN sensitive strains were precultured in YPD media for 24 h at 30°C. The OD_600_ was adjusted to 1.0 and serially diluted to 1:10, 1:50: 1:100: 1:500, and 1:1,000, and 5 µL of each dilution was spotted onto YPD agar plates without drug zero, and with 5.0, 6.35, 7.62, 9.0, and 10.0 mM of QXN. The plates were incubated at 30°C for 48 h, and results were documented using the Bio-Rad Chemi-Doc MP Imaging system 2000.

### Mutant construction

The strains were constructed using the one-step gene targeting method as described previously by Baker Brachmann et al. (1998) using the following primers:

*MFT1*-F: 5′ATG​CCT​CTG​TCA​CAA​AAA​CAA​ATA​GAC​CAA​GTT​AGA​ACC​AAA​GTG​CAC TAA​GAT​TGT​ACT​GAG​AGT​GCA​C 3′

*MFT1*-R:5′TCATTTTACTTCTTCAACAGAGGAAGACGCACTAAAATCGCTCTGTGCG GTATTTCACACCGC 3′

The verification primers are as follows:

*MFT1* Verifications F: 5′ CAA​GGC​CGA​GAT​ACA​TAG​TTC​CGC​GG 3′

*MFT1* Verifications R: 5′ AAG​GTG​ACG​CAC​CAT​ACA​GAA​CTT​GCT​C 3′. Briefly, the *MFT1-F* and *MFT1-R* primers bearing the universal sequence were used for PCR amplification from the pRS306 plasmid the *URA3* gene (Baker et al. #15). The resulting PCR fragment was introduced into the parent strain BY4741 and the isogenic *ddc1Δ*::*KANMX* strain by the standard lithium acetate method and selected for Ura + colonies. Ura + colonies carrying the *mft1Δ*::*URA3* were verified using the verification primers.

### QXN and HU sensitivities of the single and double mutant

For the sensitivities towards QXN, spot test (as described above) and growth curve assays were performed. For the growth curve, the cells were cultured overnight in YPD media then the OD_600_ was adjusted to ∼0.2 with fresh YPD media in a 96-well plate. Drugs were added to the cells at the indicated concentrations and growth was monitored for 24 h in a TECAN plate reader with shaking. The temperature was set to 30°C and the OD was taken every 2 h. The result was plotted as a graph of OD_600_ against the time (Sulaiman et al., 2022). For HU sensitivity, the spot test was performed as above. The experiments represent three independent repeats.

### Cell cycle analysis

The cell cycle analysis was performed following the protocol described by Haase and Reed, 2002; Haase (2003). Briefly, strains were grown overnight. The following day, cultures were adjusted to an optical density (OD 600) of ∼1.0 and treated with QXN (9 mM) for 4 h or left untreated. The cells were washed and left to recover for 0, 30, 60, and 120 min. Post-treatment cells were washed with distilled H_2_O twice and centrifuged. Pellets were resuspended in 70% ethanol for 30 min for fixation. The cells were washed and treated with 100 μg/mL RNase at 37°C for 16 h. The following day, the cells were washed and resuspended in 1 μg/μL proteinase K at 50°C for 30 min. The cells were washed with PBS and stained with 2.5 μg/mL propidium iodide for 20 min and analyzed on BD Fortessa X-20 flow cytometer. The data were analyzed by FlowJo software.

## Results

### Determination of QXN drug concentration that inhibits the growth of the wild-type yeast strain BY4741

We first determined the effective concentration of QXN that would kill 50% (IC_50_) of the parental strain BY4741 that was used for creating the haploid gene-deletion mutant collection. Briefly, the BY4741 strain was grown overnight, OD _600_ was adjusted to 1.0 followed by acute treatment for 4 h with increasing concentrations of QXN in liquid culture, and allowed to grow overnight in fresh media. The final OD _600_ was measured and plotted against QXN concentrations. The result indicated that the growth of the cells was severely impeded by increasing concentrations of QXN, showing a significant decrease at 11.5 mM and more drastically at 13.5 mM (Supplementary Figure S1B). The QXN IC_50_ for the BY4741 strain was determined to be ∼12 mM. In a separate experiment, the cells were adjusted to OD _600_ of ∼0.2 in liquid YPD and incubated in the absence and presence of increasing concentrations of QXN. The final OD _600_ was determined after 24 h of growth. Under this chronic drug treatment, the growth of the cells was significantly delayed (Supplementary Figure S1C). At least, 50% growth was observed at 16 h when the cells were exposed to 12.5 mM of QXN as compared to the control (Supplementary Figure S1C). Only slight growth of BY4741 was observed at 16 h with 15 mM QXN (Supplementary Figure S1C). Thus, the strain BY4741 is unable to tolerate the toxic effects of QXN concentrations greater than 15 mM.

We next determined the QXN concentrations that would effectively prevent the growth of strain BY4741 on solid YPD media to establish a suitable condition to perform the drug screening analysis of the haploid mutant collection. Briefly, four independent cultures of the BY4741 strain were grown overnight and the next day the OD _600_ was adjusted to 1.0 before serially diluted and spotted onto plates with increasing concentrations of QXN (Supplementary Figure S2A). A 100-fold dilution of each culture grew on plates with 9.5 mM QXN, but very poorly on plates with 11.5 mM QXN, suggesting that this later dose is lethal to the strain, and mutants that are sensitive to the drug are not expected to grow at 9.5 mM QXN. Similarly, when cells of OD _600_–1.0 were diluted 1000-fold and plated directly onto plates with increasing concentrations of QXN, the viability of the cells sharply decreased (Supplementary Figure S2B; and the quantification shown in Supplementary Figure S2C).

### Isolation of QXN-sensitive strains from the yeast haploid mutant collection

**FIGURE 1:**
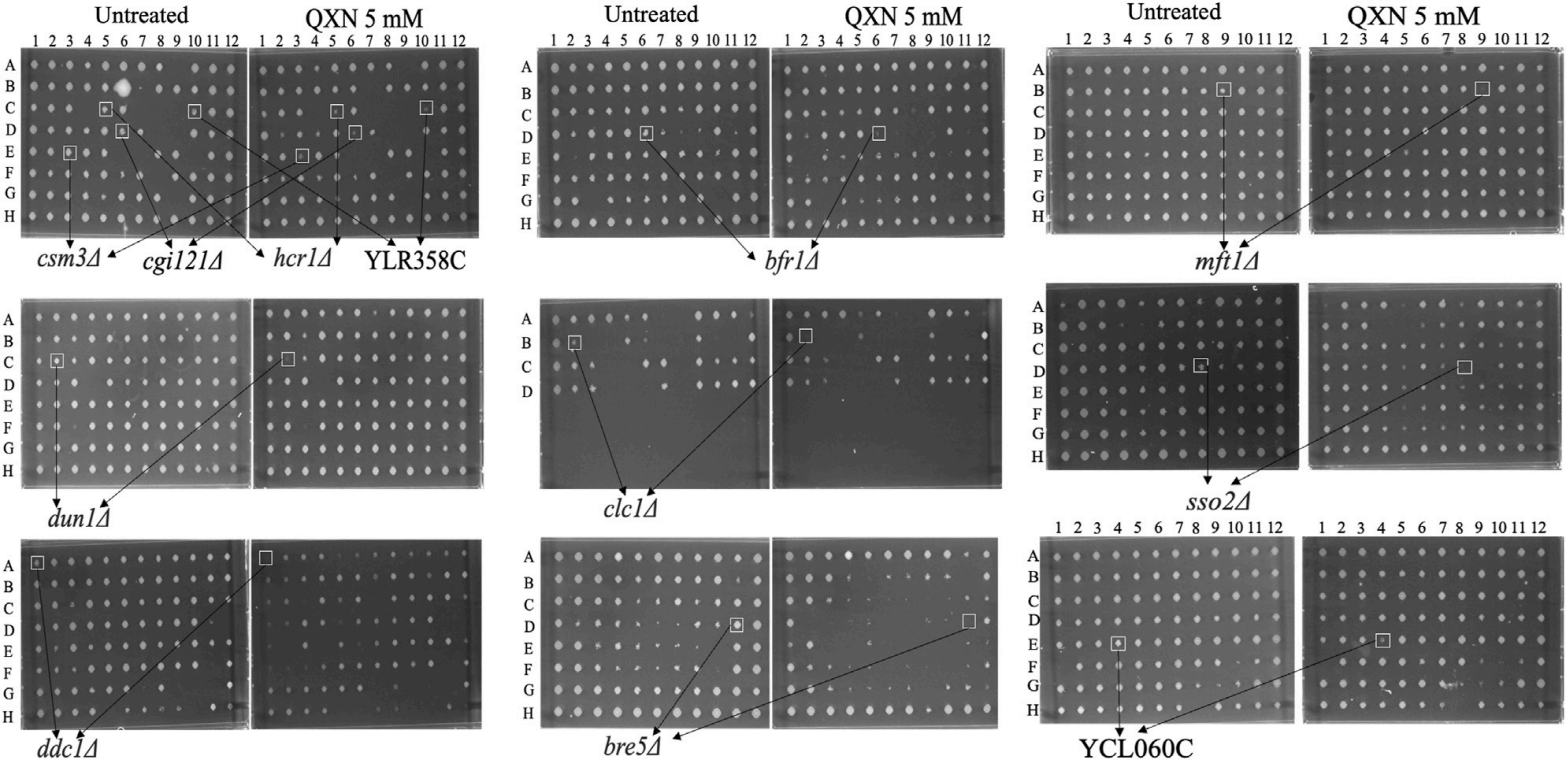
Identification of the QXN-sensitive yeast mutants from the haploid mutant collection. Briefly, frozen stocks of the haploid collection were allowed to grow overnight in YPD media and subjected to the robotic screen as outlined in the illustration shown in Supplementary Figure S3. Each spot represents a mutant from the collection that was spotted on solid YPD plates without and with 5 mM of QXN. Following 2 days of growth at 30°C, the plates were photographed. The opened square highlighted the mutants (e.g., *ddc1Δ* and *mft1Δ*) that are sensitive to QXN and the arrows point to the assigned gene name from the *Saccharomyces* Genome Database. The data are representative of three independent High-Throughput screens.

**FIGURE 2:**
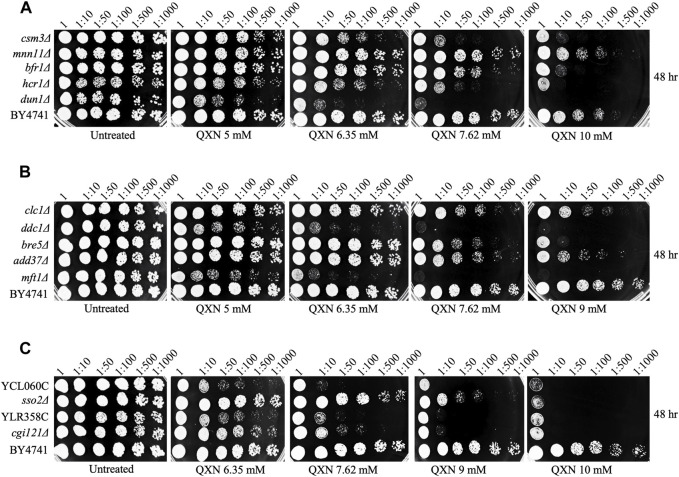
Spot test analysis confirms the sensitivity of the mutants to QXN. Cells were grown overnight, serially diluted and spotted onto plates with the indicated concentrations of QXN. The plates shown in panels **(A–C)** were photographed after 48 h incubation at 30°C. The QXN-sensitive strains are compared to the parent strain BY4741. The experiment was independently repeated at least three times.

The yeast haploid mutant collection consisted of 4,852 strains representing single gene deletion for nearly 4,000 genes (Aouida et al., 2004). Briefly, the mutant collection was allowed to grow overnight in YPD liquid culture in 96-well plates, and the strains were pinned onto YPD solid plates without and with increasing concentrations of QXN up to 19.2 mM, followed by growth for 48 h at 30°C before scoring for the most sensitive strains to the drug (see illustration in Supplementary Figure S3, and Materials and Methods). The entire haploid mutant collection was screened independently three times and from all the analyses, 12 mutants were found to be very sensitive to QXN at concentrations lower than 9.5 mM (Figure 1 and Supplementary Table S1). These mutants were retested and three gene-deletion mutants *dun1Δ*, *ddc1Δ*, and *mft1Δ* showed severe sensitivity to QXN. The *DUN1* and *DDC1* genes have been shown to encode proteins involved in cell cycle function, while *MFT1* encodes a protein that belongs to the THO complex involved in transcription (Supplementary Table S1). Two of the sensitive strains were deleted for genes with no known biological function (Figure 1 and Supplementary Table S1), and the other mutants are defective in physiological pathways that affect, for example, vacuolar function (Cohen et al., 2003). The sensitivities of these 12 mutants were independently confirmed using spot test analysis (Figure 2), and the data were consistent with the initial screen performed by the robot.

### *ddc1Δ* and *mft1Δ* function in separate pathways

**FIGURE 3:**
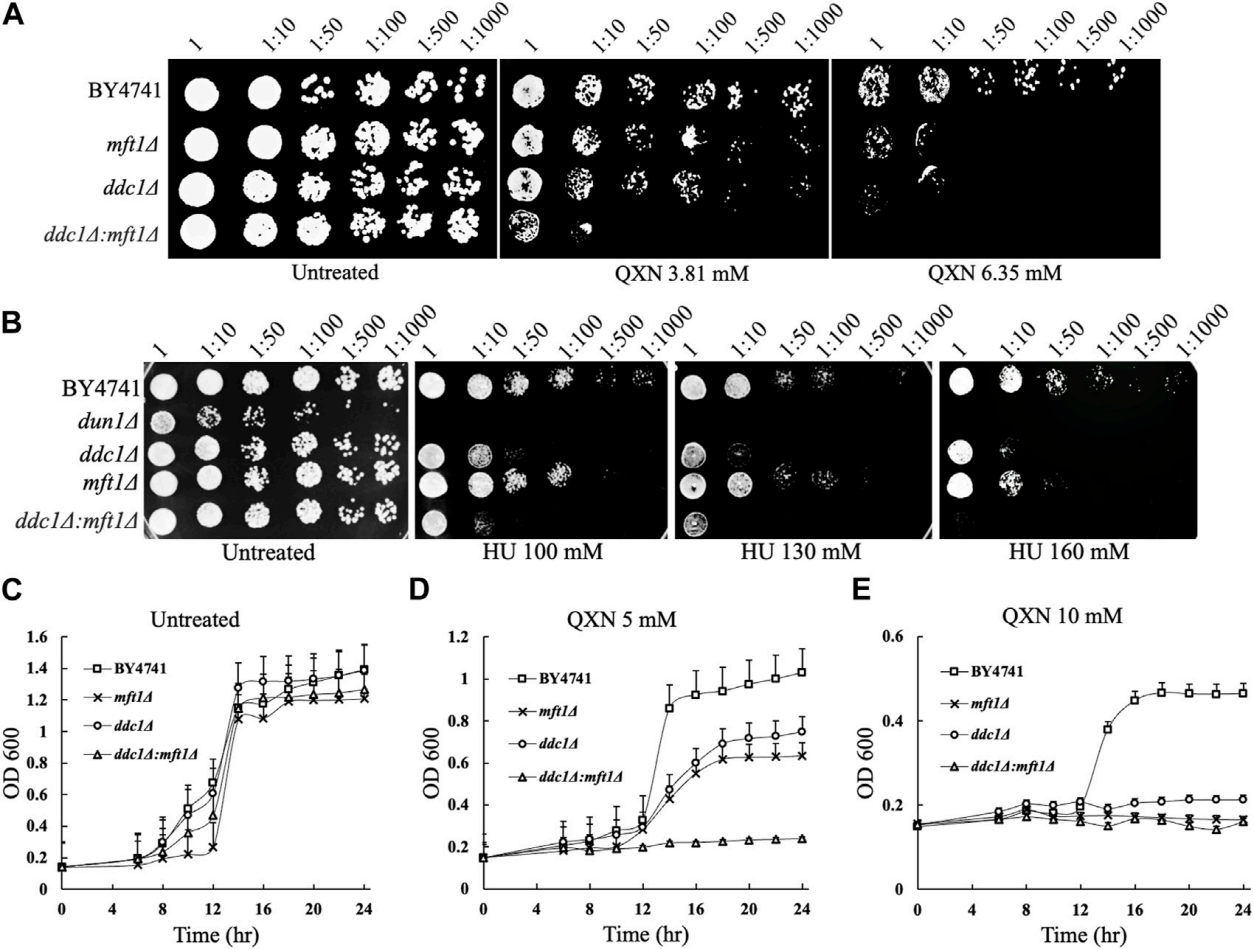
Comparison of the sensitivity of the parent, the single mutants *mft1Δ* and *ddc1Δ* and the double mutant *ddc1Δ mft1Δ* towards QXN and HU. **(A,B)**, The indicated strains were grown overnight, serially diluted and spotted onto plates containing QXN and HU, respectively. The data is representative of two independent experiments. The plates were photographed after 48 h incubation at 30°C. BY4741 is the parent. **(C–E)**, Cells from overnight cultures were adjusted to OD_600_ of ∼0.15 and allowed to grow in the absence (panel C) and presence (panels D and E) of the indicated QXN concentrations. The OD_600_ was taken at the indicated time and plotted. The results shown are the averages of three independent experiments and the error bars indicate the standard deviation.

Since the *dun1Δ*, *ddc1Δ,* and *mft1Δ* mutants were the most sensitive to QXN, besides the two mutants with unknown functions, and Dun1 and Ddc1 are involved in cell cycle arrest, we examined whether Mft1 would be implicated in the cell cycle process, as such role has not been assigned to this protein. We first independently examined whether recreating the *mft1Δ* mutant would result in sensitivity to QXN. Indeed, deletion of the *MFT1* gene in the parent strain BY4741, using one-step gene targeting and confirmed by PCR analysis, resulted in new *mft1Δ* mutants, which showed nearly the same level of sensitivity to QXN as the screened mutant (Figure 3A). We next checked whether Mft1 and Ddc1 belong to the same pathway. As such, the *MFT1* gene was deleted in the single *ddc1Δ* mutant to produce the double deletion *ddc1Δ mft1Δ* mutant. We tested the sensitivity of the single mutant against the double mutant and the result showed that the single *mft1Δ* and the single *ddc1Δ* mutants were as sensitive to QXN as compared to the parent strain BY4741 (Figure 3A). In contrast, the *ddc1Δ mft1Δ* double mutant was extremely sensitive to QXN (Figure 3A). Consistent with this observation, the *mft1Δ* and the *ddc1Δ* single mutants showed the same extent of growth inhibition in liquid culture in the presence of 5 mM QXN, while the double mutant did not grow, as compared to the parent and under no drug treatment (Figure 3D vs.Figure 3C). These findings strongly suggest that both Ddc1 and Mft1 function in separate pathways.

The *dun1Δ* mutant is known to be defective in S phase arrest and this mutant is sensitive to hydroxyurea (HU) (Palou et al., 2010), an agent that inhibits the ribonucleotide reductase needed to maintain the pool of dNTP for DNA synthesis. Since the *ddc1Δ* and *mft1Δ* mutants showed nearly the same level of sensitivity to QXN as the *dun1Δ* single mutant (Figure 3A), we checked whether the *ddc1Δ* and *mft1Δ* mutants would be as sensitive to HU. While the *dun1Δ* mutant displayed extreme sensitivity to HU, the *ddc1Δ* and the *mft1Δ* single mutants were also sensitive to HU, but to a lesser extent (Figure 3B). However, the *ddc1Δ mft1Δ* double mutant was more sensitive to HU than either the *ddc1Δ* or the *mft1Δ* single mutant, suggesting that both Ddc1 and Mft1 contribute to the S phase arrest.

### The *mft1Δ* mutant is defective in G2/M arrest in response to QXN exposure

**FIGURE 4:**
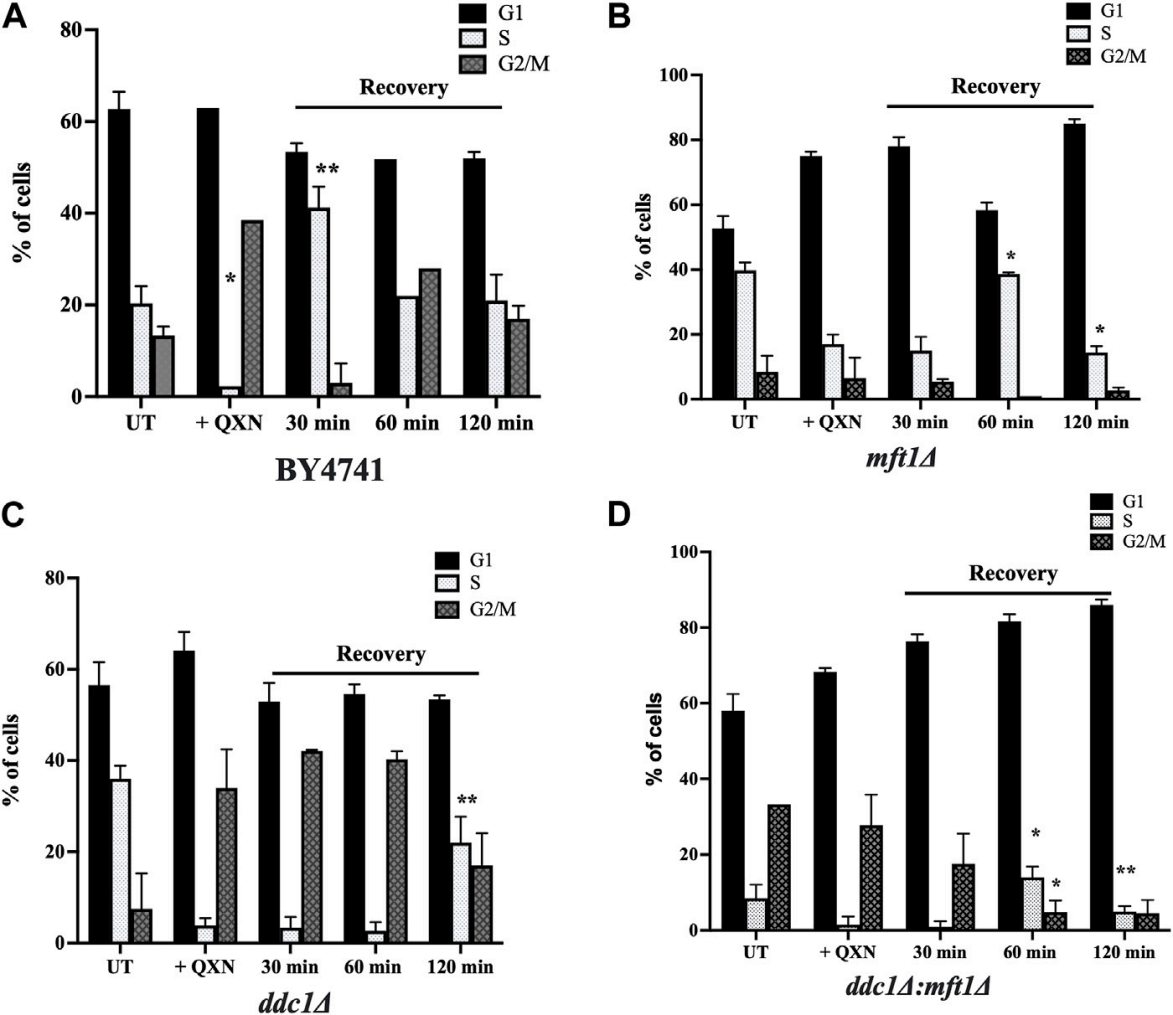
Cell cycle analysis of the parent and the indicated mutants in response to QXN and following recovery. The exponentially growing parent and mutant strains were untreated and treated with QXN (9 mM for 4 h), and the cells were washed and allowed to grow in fresh media without QXN. Samples were taken at 0, 30, 60 and 120 min as recovery time for FACS analysis as described in the Materials and Methods. **(A–D)**, The percentage of cells in the indicated phases for the untreated, treated, and post-treatment (recovery) for the parent and the mutants. Error bars represent mean ± SD. *p < 0.05, **p < 0.01. See Supplementary Figure S4 for the graphic representation of the cell cycle profiles for the indicated strains.

We examined whether QXN would trigger cell cycle arrest in a specific phase in the parent cells and whether this arrest would be disrupted by the loss of Ddc1 or Mft1 or both. Briefly, the asynchronous population of the parent, the single mutants *ddc1Δ* and *mft1Δ*, and the double mutant *ddc1Δ mft1Δ* were untreated and treated for 4 h with QXN (9.5 mM). The cells were washed and allowed to recover in fresh media without QXN. Samples were taken at 0, 30, 60 and 120 min, and processed for cell cycle analysis using the Sytox Green dye (Haase, 2003). In response to QXN, and at 0 mins recovery time, the parent showed an increase in the number of cells in the G2/M phase as compared to the asynchronous population of untreated cells (Figure 4A). Following 30 min of recovery, the parent displayed an increase in the number of cells in S phase arrest and by 120 min resumed normal cycling (Figure 4A). These observations suggest that the dose of QXN is capable of inducing toxic DNA lesions thereby triggering cell cycle arrest in both the S and G2/M phases. The *ddc1Δ* mutant showed the same extent of G2/M arrest as the parent following treatment with QXN, and displayed no S phase arrest during the 30-min or 60-min recovery, but retained the G2/M arrest and by 120 min remedied the QXN-induced lesions (Figure 4C). Thus, it would appear that the *ddc1Δ* mutant lacks the S phase arrest required to process QXN-induced DNA lesions. In contrast, the *mft1Δ* mutant showed no major G2/M arrest upon treatment with QXN, as compared to the parent or the *ddc1Δ* mutant, but showed a delayed S phase arrest that appeared during the 60-min recovery phase (Figure 4B). The *mft1Δ* mutant failed to recover the normal cell cycle progression after 120 min, as compared to the parent (Figure 4B vs. Figure 4A). We interpret this observation to suggest that the *mft1Δ* mutant is defective in activating the G2/M phase arrest in response to QXN treatment. In the case of the *ddc1Δ mft1Δ* double mutant, it showed a synergistic effect in response to the QXN treatment with no S phase arrest at 30 min and a weakened G2/M arrest at 60 min, with most of the cells accumulating in the G1 phase (Figure 4D). Based on the above findings, it would appear that Mft1 plays a role in triggering the G2/M phase arrest upon exposure to QXN.

## Discussion

In this study, we identified 12 gene-deletion mutants from the *S. cerevisiae* haploid mutant collection that displayed varying sensitivity to QXN. However, we found none of the mutants in the entire collection to be resistant to the drug. It is possible that more than one gene might be involved in contributing to the QXN-resistance phenotype or such a resistant gene is essential for viability. Nonetheless, of the QXN-sensitive mutants, three of these (*dun1Δ*, *ddc1Δ*, and *mft1Δ*) displayed hypersensitivity to QXN, suggesting that the Dun1, Ddc1, and Mft1 proteins perform a major role in the response to the drug. Dun1 is a serine/threonine protein kinase that operates in the S phase checkpoint, for example, when the replication fork is obstructed. Dun1 also has a well-defined role in upregulating the levels of the dNTPs pool (Gershon and Kupiec, 2021). It induces transcription of the ribonucleotide reductase genes RNR2, RNR3 and RNR4 and triggers the cytoplasmic-nuclear redistribution of the ribonucleotide reductase Rnr2-Rnr4 upon genotoxic stress. Dun1 can phosphorylate several proteins such as the DNA repair protein Rad55, the superoxide dismutase Sod1, and the repressor Crt1 that inhibits ribonucleotide reductase gene expression as well as the inhibitor, Sml1, of Rnr1. In addition, Dun1 is required to regulate telomere length, as well as late firing of origins of replication (Gershon and Kupiec, 2021). Thus, the sensitivity of the *dun1Δ* mutant to QXN can be explained by a defect in the S-phase checkpoint, causing damaged DNA such as incomplete replicated DNA to enter the G2/M phase.

Ddc1 is a DNA damage checkpoint protein that inhibits the cell cycle in response to unrepaired DNA lesions (Hong and Roeder, 2002). The observation of the S phase arrest in the parent strain in response to QXN treatment, and not in the *ddc1Δ* mutant, is consistent with QXN causing replication intermediates requiring the function of Ddc1 to trigger S phase arrest. In the absence of Ddc1, the QXN-induced DNA lesions would be expected to activate the G2/M phase arrest as observed (Figure 4). The Ddc1 protein forms a heterotrimeric complex with Rad17 and Mec3 (Rad17-Mec3-Ddc1), also referred to as the yeast counterpart of the human 9-1-1 complex. The Rad17-Mec3-Ddc1 complex forms a ring structure, similar to the PCNA-like checkpoint complex, and plays a role in ensuring the integrity of the DNA before DNA synthesis or separation of the daughter chromosomes. This complex is loaded onto the site of DNA damage to form nuclear foci, aided by its interaction with the RAD24-RFC checkpoint clamp loader, which then activates the Mec1 kinase for cell cycle arrest during the G1 and G2/M phases (Liu, 2019). The activation of the Mec1 kinase requires the function of Ddc1, which has a C-terminal tail that is heavily phosphorylated, and implicated in the recruitment of various DNA damage response proteins. Thus, the defective S phase arrest observed by the *ddc1Δ* mutant in response to QXN could be explained by the failure of the mutant to activate the Mec1 kinase (Liu, 2019). Interestingly, while Ddc1 is involved in the interaction with Rad17, our screen did not detect the *rad17Δ* mutant as one that is sensitive to QXN or any member of the RAD24-RFC complex, suggesting that Ddc1 may perform a specific function in response to QXN. Based on our findings, we anticipate that the human counterpart, the 9-1-1 complex, of the yeast Rad17-Mec3-Ddc1 complex would play a pivotal role in QXN response.

In the case of Mft1, it has not been previously shown to be involved in cell cycle arrest, although little is known regarding its exact function. Mft1 belongs to a complex called THO that is involved in transcription elongation and mRNA export (Chávez et al., 2000). The THO complex consists of four proteins Hpr1, Mft1, Tho2 and Thp2. A defect in this THO complex impedes transcription such that it generates DNA-RNA hybrid (R loops), that is, displaced complementary single-stranded DNA, which can stimulate lethal transcription-associated recombination leading to genomic instability (Gómez-González et al., 2009). These R loops can perform specific functions such as DNA replication. However, R loops can cause DNA breaks leading to recombination and chromosomal rearrangements (García-Muse and Aguilera, 2019). Since the additional members of the THO complex were not recovered as QXN-sensitive mutants, it is reasonable to propose that Mft1 could have a distinct role in the response to QXN. The observation that the *mft1Δ* mutant is unable to efficiently arrest the cells in the S phase and showed no G2/M phase arrest, as compared to the parental strain in response to QXN, suggests that Mft1 performs a distinct role from Ddc1. We believe that Mft1 is required to trigger a G2/M phase arrest to allow the cells to process R-loop intermediates that might be created by QXN leading to DNA double-strand breaks. We conclude that Mft1 plays a major role in triggering G2/M phase arrest, while Ddc1 is required for activating the S phase arrest in response to QXN exposure. Thus, cells devoid of both Mft1 and Ddc1 are expected to be profoundly more sensitive to QXN, as opposed to the individual mutant (Figure 3D). So far, a counterpart of Mft1 does not appear to exist in human cells, although it is conserved in other yeast species indicating that Mft1 role may be specific for these fungi.

It is noteworthy that although our genome-wide screen identified other defective gene functions that are related to Golgi trafficking, vacuolar detoxification, and proteolysis, the major effects caused by QXN appear to involve processes that maintain the integrity of the DNA. As such, we believed that QXN could be an effective drug to target tumours that are defective in cell cycle arrest.

## Scope statement

The drug quinoxaline and its derivatives have broad applications, in particular controlling bacterial, fungal and viral infections, as well as possessing antitumor activities. Quinoxaline can induce reactive oxygen species and is believed to damage various macromolecules including the DNA. However, the exact mechanism by which this drug causes toxicity is not known. We set out to search for molecular factors that would interfere with the responses of the budding yeast *S. cerevisiae* to quinoxaline. We exploited the library of yeast haploid gene-deletion mutants and identified at least 12 with varying sensitivity to quinoxaline. We showed that one of the hypersensitive mutants lacks the *MFT1* gene with no previous role in quinoxaline response. We report for the first time that quinoxaline exerts its toxic effects by inducing cell cycle arrest and that Mft1 is required to mediate this process. The genetic characterization presented herein paves the way to determine precisely whether oxidative DNA lesions generated by reactive oxygen species produced by quinoxaline are responsible for cell cycle arrest. As such, any clinical application of quinoxaline and its derivatives should entail targeting cancer cells with defective cell cycle arrest.

## Data Availability

The original contributions presented in the study are included in the article/Supplementary Material, further inquiries can be directed to the corresponding authors.

## Supplementary material

SUPPLEMENTARY FIGURE S1 Growth rate analysis of strain BY4741 in response to acute and chronic exposure to quinoxaline (QXN). **(A)**, Chemical Structure of quinoxaline. **(B)**, Cells were grown overnight and the next day the OD_600_ was adjusted to OD 1.0 followed by acute treatment for 4 h with increasing concentrations of QXN in liquid culture. The cells were washed, diluted to OD_600_ of ∼0.2, and allowed to grow overnight in fresh media. The final OD_600_ after overnight growth was measured and plotted against QXN concentrations. **(C)**, Cells were adjusted to OD_600_ of ∼0.2 in liquid YPD and incubated in the absence and presence of increasing concentrations of QXN for overnight treatment. The final OD_600_ was determined after 24 h of growth. The results shown in panels B and C are the averages of three independent experiments and the error bars indicate the standard deviation.

SUPPLEMENTARY FIGURE S2 Determination of the QXN concentrations needed to perform the genome-wide screen. **(A)**, Four independent colonies of strain BY4741 were grown overnight, serially diluted and spotted onto solid YPD agar plates containing the indicated concentrations of QXN ranging from 0 to 15.5 mM. The plates were incubated for 48 hrs at 30°C and the images were taken using Bio-Rad Chemi-Doc MP Imaging system. **(B)**, Cells adjusted to OD _600_ of ∼1.0, treated for 4 h with the indicated concentrations of QXN, diluted 1000-fold and plated directly onto plates without QXN. The surviving colonies were scored after 48 h of growth at 30°C. The colonies of the plated yeast cells were counted using Image J. Data are mean SEM of n=3, and significance is assumed at *p** < 0.05. **(C)**, Plot of the surviving fraction of the cells following treatment with QXN.

SUPPLEMENTARY FIGURE S3 Illustration of the screening approach to identify QXN-sensitive mutants. Frozen stocks of the haploid yeast mutant collection were cultured overnight into 96-well plates in YPD liquid media. The following day the cells were pinned by the aid of a ROTOR HDA onto plates without and with increasing concentrations of QXN. Plates were incubated at 30°C for 48 to 72 h and photographed using the Bio-Rad Chemi-Doc MP Imaging system. A representative plate is shown and a fading spot is indicative of a QXN-sensitive mutant.

SUPPLEMENTARY FIGURE S4 Cell cycle profiles of the parent and the indicated mutants in response to QXN and post-treatment recovery. The cell cycle analysis was performed as described in Figure 4, and the percentage of cells in the indicated phases for the untreated, treated, and post-treatment (recovery) were derived from the graphic profiles. The representative profiles correspond to the untreated cells, treated cells without recovery (0 min), and treated cells recovered for 120 min. The G1 phase is shown in purple, the S phase in yellow, and G2/M in green.
