# Supplementary figures and images for: Mft1, identified from a genome-wide screen of the yeast haploid mutants, mediates cell cycle arrest to counteract quinoxaline-induced toxicity
Source: Front Genet. 2024 Jan 12;14:1296383. doi: 10.3389/fgene.2023.1296383 (PMC10811161; doi:10.3389/fgene.2023.1296383)

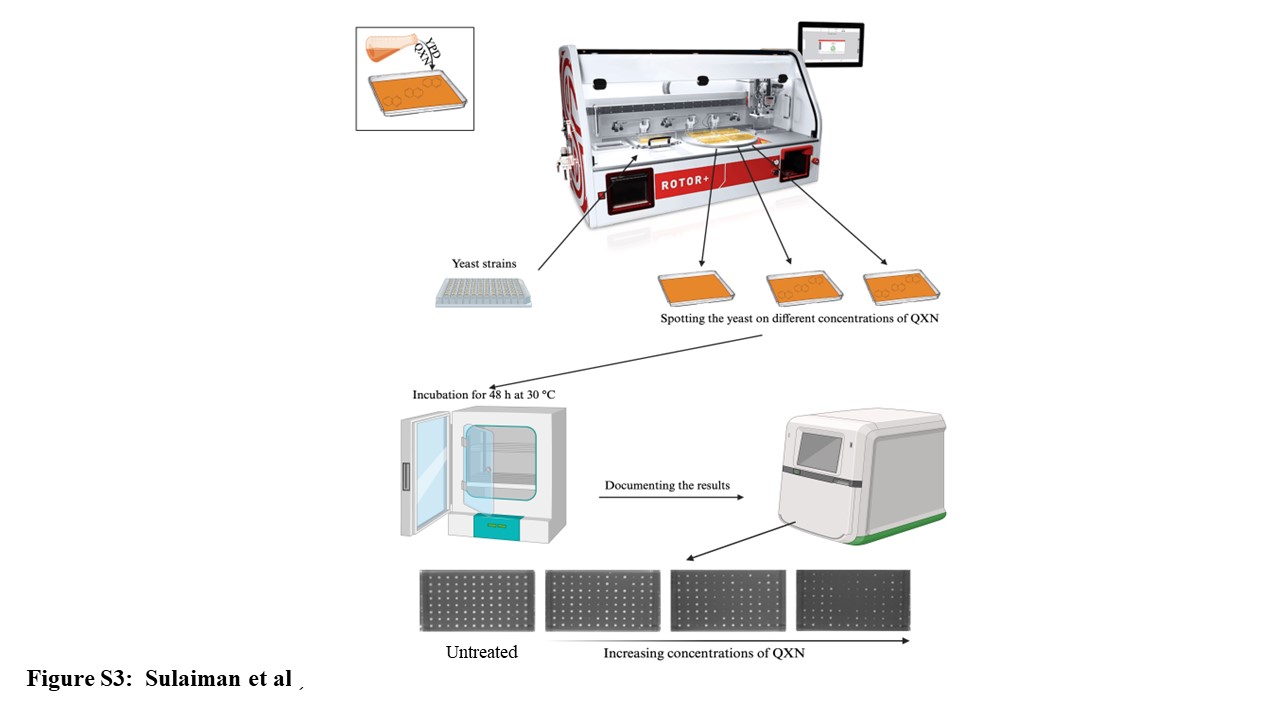

Supplement: Supplementary file 1 [file Image3.JPEG]

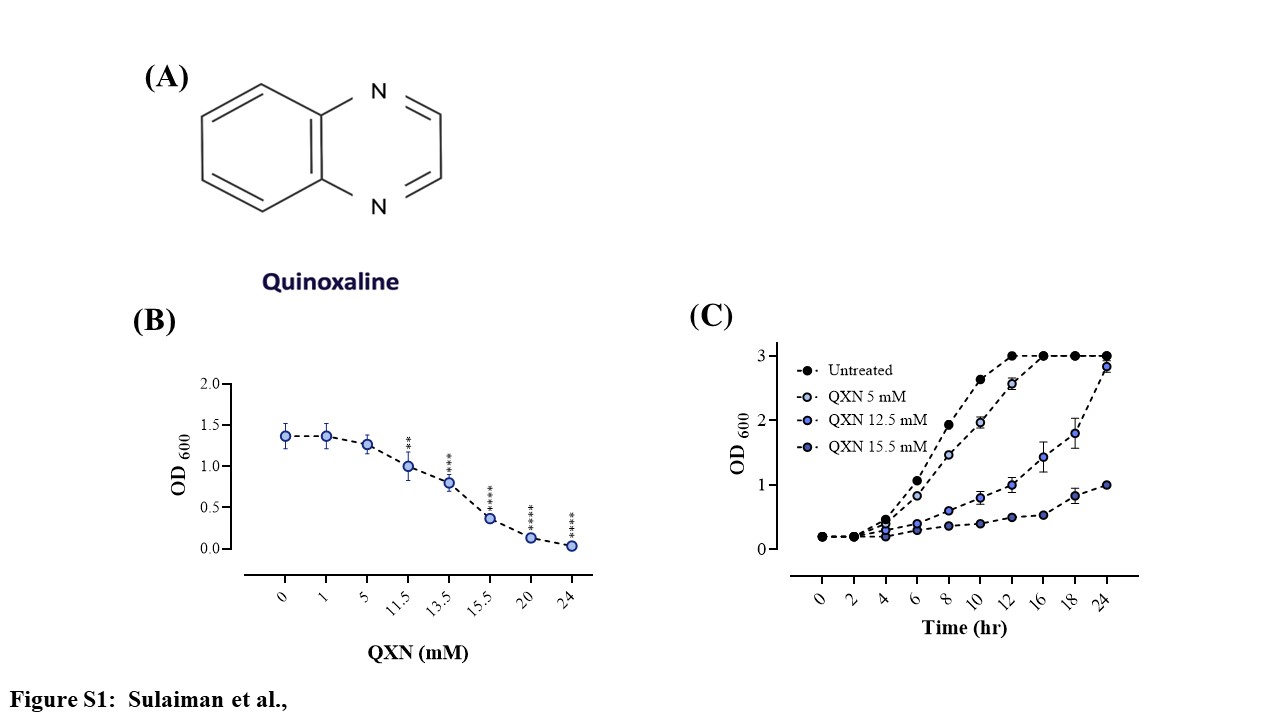

Supplement: Supplementary file 3 [file Image1.JPEG]

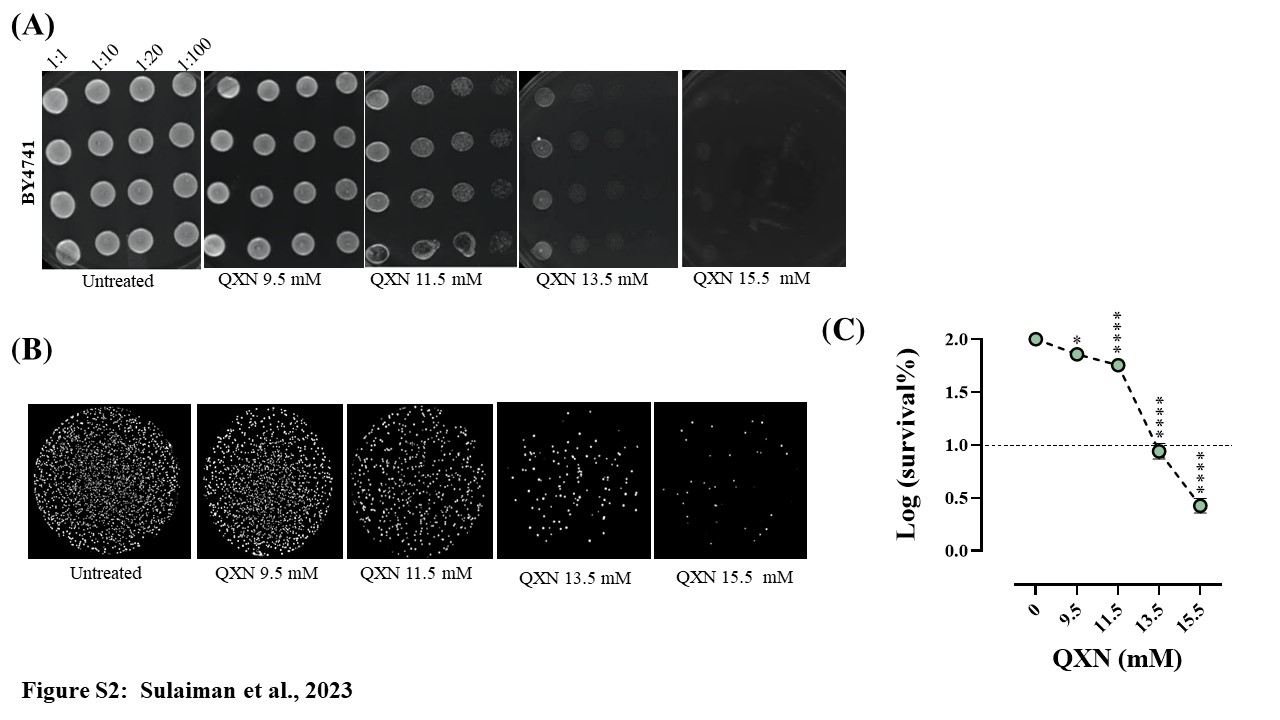

Supplement: Supplementary file 4 [file Image2.JPEG]

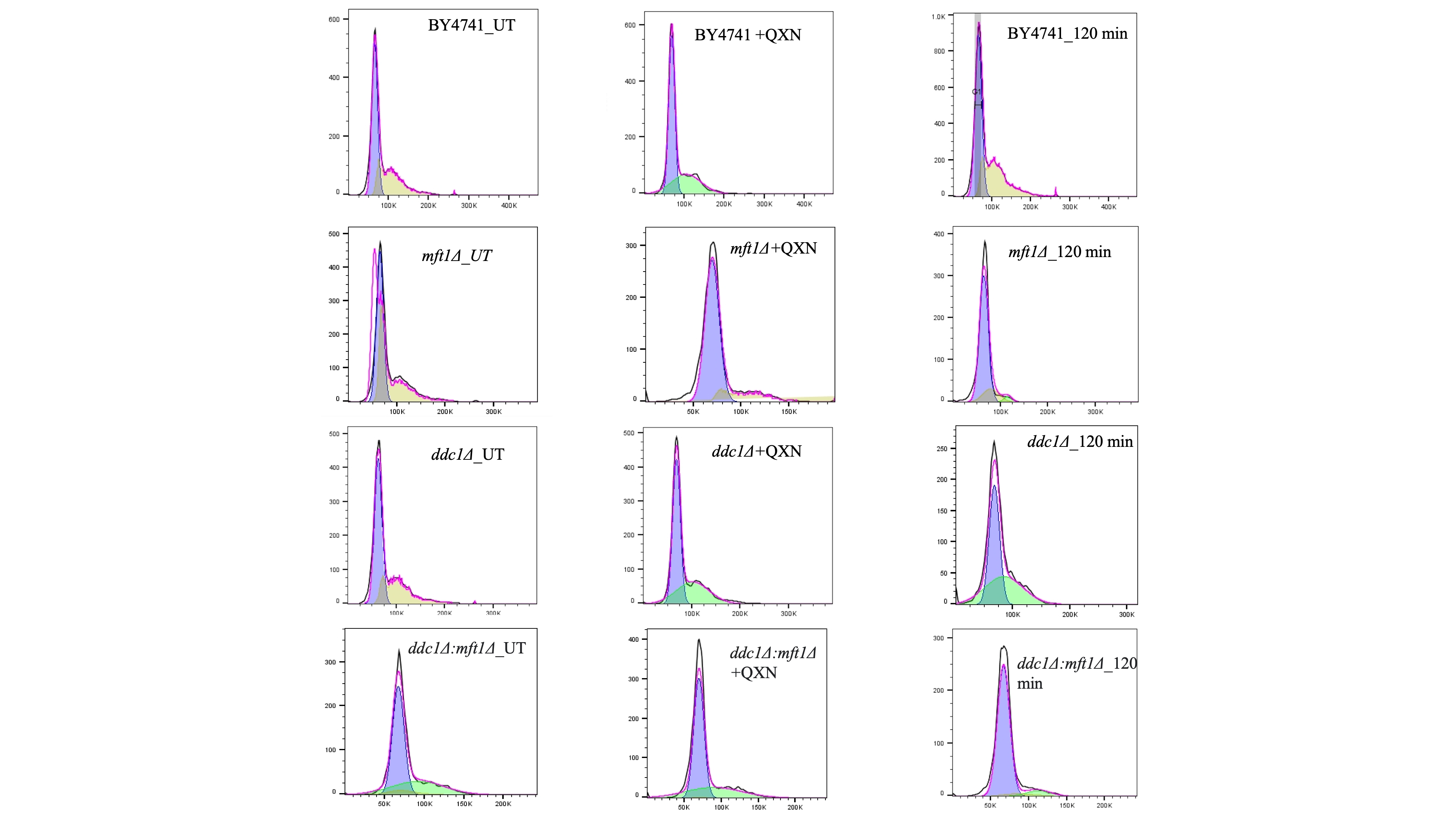

Supplement: Supplementary file 5 [file Image4.TIFF]
