# Supplementary material for: Mft1, identified from a genome-wide screen of the yeast haploid mutants, mediates cell cycle arrest to counteract quinoxaline-induced toxicity
Source: Front Genet. 2024 Jan 12;14:1296383. doi: 10.3389/fgene.2023.1296383 (PMC10811161; doi:10.3389/fgene.2023.1296383)
Supplement: Supplementary file 2 [file Table1.DOCX]

| **Table 1. List of gene-deletion mutants with varying sensitivity towards QXN and the known defective biological function.** | | | |
| --- | --- | --- | --- |
| ORF | Gene | Sensitivity | Functional role (from SGD) |
|  | BY4741 | + | Parent |
| YMR048W | *csm3Δ* | +++++ | Replication fork associated factor; required for stable replication fork pausing; component of the DNA replication checkpoint pathway; required for accurate chromosome segregation during meiosis; forms nuclear foci upon DNA replication stress. |
| YOR198C | *bfr1Δ* | +++++ | Component of mRNP complexes associated with polyribosomes; involved in localization of mRNAs to P bodies; implicated in secretion and nuclear segregation; multicopy suppressor of BFA (Brefeldin A) sensitivity. |
| YLR192C | *hcr1Δ* | ++++++ | eIF3j component of translation initiation factor 3 (eIF3); dual function protein involved in translation initiation as a substoichiometric component (eIF3j) of eIF3; required for 20S pre-rRNA processing; required at post-transcriptional step for efficient retrotransposition; absence decreases Ty1 Gag:GFP protein levels; binds eIF3 subunits Rpg1p, Prt1p and 18S rRNA; eIF3 also involved in programmed stop codon read through; human homolog EIF3J can complement yeast hcr1 mutant. |
| YDL101C | *dun1Δ* | +++++++ | Cell-cycle checkpoint S/T protein kinase; required for transient G2/M arrest after DNA damage, damage-induced transcription, and nuclear-to-cytoplasmic redistribution of Rnr2p-Rnr4p after genotoxic stress and iron deprivation; phosphorylates repair protein Rad55p, transcriptional repressor Sml1p, superoxide dismutase, and ribonucleotide reductase inhibitors Crt1p and Dif1p; functions in the Mec1p pathway to regulate dNTP pools and telomere length; postreplicative repair role. |
| YGR167W | *clc1Δ* | ++ | Clathrin light chain; subunit of the major coat protein involved in intracellular protein transport and endocytosis; regulates endocytic progression; thought to regulate clathrin function; the clathrin triskelion is a trimeric molecule composed of three heavy chains that radiate from a vertex and three light chains which bind noncovalently near the vertex of the triskelion. |
| YPL194W | *ddc1Δ* | +++++++ | DNA damage checkpoint protein; part of a PCNA-like complex required for DNA damage response, required for pachytene checkpoint to inhibit cell cycle in response to unrepaired recombination intermediates; potential Cdc28p substrate; forms nuclear foci upon DNA replication stress. |
| YNR051C | *bre5Δ* | +++++ | Ubiquitin protease cofactor; forms deubiquitination complex with Ubp3p that coregulates anterograde and retrograde transport between the ER and Golgi compartments, deubiquitinating COPII and COPI vesicle coat constituents, Sec23p and Sec27p; involved along with Ubp3p in the steady-state retention of Golgi membrane proteins, such as glycosyltransferases; null is sensitive to brefeldin A. |
| YML062C | *mft1Δ* | +++++++ | Subunit of the THO complex; THO is a nuclear complex comprised of Hpr1p, Mft1p, Rlr1p, and Thp2p, that is involved in transcription elongation and mitotic recombination; involved in telomere maintenance. |
| YCL060C | Unknown | ++++++ | Merged open reading frame; does not encode a discrete protein; YCL060C was originally annotated as an independent ORF, but as a result of a sequence change, it was merged with an adjacent ORF into a single reading frame, designated YCL061C. |
| YMR183C | *sso2Δ* | +++ | Plasma membrane t-SNARE; involved in fusion of secretory vesicles at the plasma membrane; syntaxin homolog that is functionally redundant with Sso1p; SSO2 has a paralog, SSO1, that arose from the whole genome duplication. |
| YLR358C |  | +++++ | Protein of unknown function; expressed at both mRNA and protein levels; partially overlaps ORF RSC2/YLR357W. |
| YML036W | *cgi121Δ* | +++++ | Component of the EKC/KEOPS complex; EKC/KEOPS complex is required for t6A tRNA modification and telomeric TG1-3 recombination; may have role in transcription; Cgi121p is dispensable for tRNA modification; other complex members are Bud32p, Kae1p, Pcc1p, and Gon7p; ortholog of human TPRKB. |
|  | *ddc1Δ:mft1Δ* | ++++++++ |  |
|  |  |  |  |
| Sensitivity was determined based on the spot test analysis.  The plus sign indicates the level of sensitivity towards QXN, with eight plus signs (++++++++) being the most sensitive to QXN and a single plus sign (+) being the least sensitive.  The column showing the functional role was obtained from the Sacchromyces Genome Database. | | | |
